# Optic Disc Flavoprotein Fluorescence Imaging as a Novel Method to Quantify Disease Burden in Optic Disc Drusen

**DOI:** 10.1016/j.ajo.2025.11.018

**Published:** 2025-11-17

**Authors:** RISHITA RAMA PUJARI, MIAOMIAO YU, JAMIE ZHANG, LORRAINE ALMEDA, SANGEETHABALASRI PUGAZHENDHI, PING ZHU, COLLIN RICH, HEATHER E. MOSS, SHANNON BERES, YAPING JOYCE LIAO

**Affiliations:** Department of Ophthalmology (R.R.P., M.Y., J.Z., L.A., S.P., P.Z., H.E.M., S.B., Y.J.L.), Stanford University School of Medicine, Stanford, California, USA; OcuSciences, Inc. (C.R.), Ann Arbor, Michigan, USA; Department of Neurology and Neurological Sciences (H.E.M., S.B., Y.J.L.), Stanford University School of Medicine, Stanford, California, USA

## Abstract

**PURPOSE:**

To investigate the ability of flavoprotein fluorescence (FPF) imaging to quantify disease burden in optic disc drusen (ODD).

**DESIGN:**

Cross-sectional study.

**PARTICIPANTS:**

One hundred and fifty-seven ODD eyes (94 participants, ages 7-89 years) and 69 control eyes (53 participants, ages 10-78 years).

**METHODS:**

Comprehensive examination, visual function testing, and multimodal ophthalmic imaging. Statistical analysis was performed using parametric and nonparametric tests, ANOVA, and Spearman correlation.

**MAIN OUTCOME MEASURES:**

LogMAR, static perimetry mean deviation, optic disc and macular FPF, enhanced-depth imaging optical coherence tomography (EDI-OCT), OCT peripapillary retinal nerve fiber layer (pRNFL), and macular ganglion cell complex (mGCC) thickness.

**RESULTS:**

Optic disc FPF signal corresponded with superficial and buried drusen visualized on 97 EDI-OCT B-scans. Compared with controls, ODD eyes have significantly elevated disc FPF (*P* < .0001) but no difference in macular FPF scores. Examination of age-related changes revealed stable disc FPF in controls over the first 7 decades of life. In contrast, ODD eyes exhibited elevated disc FPF within the first 2 decades of age, which increased over time and remained high after age 40. Sectoral analysis showed significantly elevated disc FPF in all quadrants in ODD compared with controls (*P* < .001). ODD eyes with visual field loss (mean deviation (MD) < −2 dB) had significantly higher disc FPF and lower pRNFL and mGCC thicknesses compared with ODD eyes without visual field loss (MD ≥ −2 dB) (*P* < .001 for all). We found a nonlinear relationship between disc FPF and MD (RMSE = 4.4271, R^2^ = 0.4504) and a negative correlation between disc FPF and pRNFL (r = −0.78) and mGCC thicknesses (*r* = −0.62). Disc FPF, pRNFL, and mGCC had high statistical power in segregating ODD eyes with and without visual field loss.

**CONCLUSIONS:**

Disc FPF is an objective imaging technique to quantify disease burden in ODD, reflecting a combination of drusen autofluorescence signal and metabolic stress from axonopathy. Disc FPF is correlated with structural and functional changes and has high predictive power of visual field loss in ODD, supporting its use as an outcome measure in prospective natural history and treatment studies in ODD.

## INTRODUCTION

OPTIC DISC DRUSEN (ODD) ARE ACELLULAR extracellular calcified concretions in the most anterior portion of the optic nerve above the lamina cribrosa, leading to optic neuropathy and vision loss.^1–3^ ODD are found in 0.3% to 2% of the general population, and the prevalence increases to 3.1% in those with a family history of ODD.^4,5^ Given the availability of noninvasive ophthalmic imaging, patients may be incidentally found to have crowded optic discs and referred for evaluation of possible papilledema or optic disc edema^6, 7^ – a common way children are diagnosed with ODD.^8^ In adults, ODD more commonly present with visual field loss, which occurs in up to 87% of ODD cases.^4,9^ The pathogenesis of ODD is unknown but may be related to local axonopathy due to metabolic stress and local ischemia, calcium phosphate dysregulation, altered axonal transport, and extrusion of calcified mitochondria, leading to osteogenic differentiation, hydroxyapatite deposition, and biomineralization.^2,9,10^

Despite the classic appearance of ODD, it is still challenging to make the correct diagnosis using fundoscopic examination and ophthalmic imaging, especially in the pediatric population. While *en face* fundus autofluorescence (FAF) imaging can readily visualize the hyperautofluorescence of ODD, it may miss deeper structures like buried drusen and may be false negative in approximately 10% of patients with ODD.^11^ The International Optic Disc Drusen Studies Consortium recommends using enhanced depth imaging-optical coherence tomography (EDI-OCT) with a 97-line scan as the gold standard for the diagnosis of ODD, because it allows visualization of the entire optic disc and is currently the only way to be sure that a patient is a true negative.^12^ On EDI-OCT B-scans, ODD exhibits characteristic signal-poor core and hyperreflective borders, which also provide a useful morphological landscape of the optic disc in ODD.^12,13^ Unfortunately, interpretation of the EDI-OCT scan of the entire optic disc requires training, is time-consuming, and still only offers a qualitative assessment of disease burden, which limits its utility in tracking disease progression and is not a suitable endpoint for clinical trials.

Endogenous flavoprotein fluorescence (FPF) imaging is a novel, non-invasive imaging modality that provides quantitative measurement of the autofluorescence signal and a functional assessment of metabolic stress. The optic nerve head and retinal ganglion cells contain a high concentration of mitochondria.^14^ Increased reactive oxygen species, which are indicative of oxidative stress, lead to mitochondrial dysfunction, optic neuropathies, and vision loss.^15–17^ Oxidized mitochondrial flavoproteins, as flavin adenine dinucleotide or flavin mononucleotide, emit green autofluorescence upon excitation by blue light, which can be imaged and quantified.^18, 19^ Although FPF imaging has been used to study several eye diseases, such as glaucoma,^20–22^ age-related macular degeneration,^23,24^ diabetic retinopathy,^14,25,26^ and retinal dystrophies,^19,27^ the potential of FPF imaging in ODD has been unexplored. The use of FPF imaging in ODD is a natural fit, given the increased optic disc autofluorescence in ODD with FAF imaging and increased FPF in optic neuropathies.^20–22^ In this study, we asked whether FPF imaging can quantify ODD autofluorescence signal and disease burden in ODD.

## MATERIALS AND METHODS

We conducted a prospective, cross-sectional study on patients with ODD and healthy controls, who were evaluated at the Byers Eye Institute at Stanford University Medical Center between May 2021 and October 2024. The study was approved by the Stanford Institutional Review Board and adhered to the Declaration of Helsinki and the Health Insurance Portability and Accountability Act. Informed written consent was obtained from all subjects following a detailed verbal discussion on the purpose of the study, and electronic health record systems (EPIC, Forum) were reviewed to collect demographic and clinical data.

### PARTICIPANTS SELECTION AND CLINICAL EVALUATION:

We prospectively enrolled 162 participants (281 eyes), of which 53 (69 eyes) were healthy controls and 109 (212 eyes) had a confirmed diagnosis of ODD by neuro-ophthalmologists. We collected demographic information (Table 1), and all participants underwent comprehensive neuro-ophthalmic assessments. Healthy control eyes were defined as those without diseases of the eye, central nervous system, or systemic diseases that may impact ophthalmic measurements. All control eyes had best corrected visual acuity equal to or better than logMAR (LogMAR) of 0.3 (equivalent to 20/40), no visual field loss, and normal OCT peripapillary retinal nerve fiber layer (pRNFL) and macular ganglion cell complex (mGCC) analyses. These controls included 22 contralateral healthy eyes from patients with unilateral non-arteritic anterior ischemic optic neuropathy (NAION) and no vision loss. ODD was defined as participants with the diagnosis of ODD by a neuro-ophthalmologist and confirmed by multimodal ophthalmic imaging, including enhanced depth imaging optical coherence tomography (EDI-OCT), the current gold standard for diagnosis of ODD.^12^ There was no exclusion based on visual acuity or visual field in the ODD group; however, inclusion required high-quality ophthalmic imaging. We excluded eyes with complications associated with ODD or syndromic features, such as NAION, central retinal artery or vein occlusions, and retinal degeneration (*N* = 29 eyes) because these changes may impact measurements of visual function or structure.

### VISUAL FUNCTION ASSESSMENTS:

We measured central vision using best-corrected visual acuity on the Snellen chart and calculated the LogMAR. We assessed peripheral vision using automated static perimetry with the Humphrey Field Analyzer (Carl Zeiss Meditech USA, Inc.) and the Swedish Interactive Thresholding Algorithm (SITA) fast or standard 24-2 strategy to calculate the mean deviation (MD). We excluded unreliable visual field tests from analysis, which were defined as those with fixation loss greater than 20% or false-positive or false-negative error rates greater than 20%.

### OPTIC NERVE AND MACULAR MULTIMODAL IMAGING ASSESSMENTS:

All participants had multimodal imaging of the posterior pole using 4 imaging instruments. (1) After pupillary dilation, we performed optic disc and macular FPF imaging with OcuMet Beacon (OcuSciences, Inc.), a confocal scanning laser ophthalmoscope with excitation bandpass centered at 471 nm (Supplementary Figure 1) and emission band filter at 530 nm, a setting designed to capture endogenous FPF signal while minimizing contamination from endogenous retinal fluorophores such as lipofuscin. We captured a 60°W x 21.5°H infrared scan of the posterior pole and a 17°W x 21.5°H FPF scan (per capture) of fields around the optic disc and macula. (2) We acquired *en face* fundus color and green-light FAF and color fundus images using Optomap ultra-widefield fundus color and autofluorescence (FAF) imaging (OptoMAP California, Optos, Inc.), which uses confocal scanning laser ophthalmoscopy with a 200° field of view. The green light FAF uses excitation at 532 nm and emission at 480 to 800 nm, with an optical resolution of 3900 × 3072 pixels and an acquisition time of 0.25 seconds per scan. (3) To visualize all buried and superficial ODD, we performed optic disc EDI-OCT 97-line scans and 6 radial scans using Spectralis HRA + OCT (Heidelberg Engineering, Heidelberg, Germany), a spectral-domain OCT instrument that acquires high-resolution 40,000 A-scans/s at an axial resolution of 7 μm. (4) To quantify optic nerve and macular structural thicknesses, we performed optic nerve head and macular analyses using Cirrus HD-OCT 5000 (Carl Zeiss Meditech USA, Inc.), a spectral-domain OCT with a superluminescent diode laser light source at 840 nm that acquires 68,000 A-scans/s and has axial resolution of 5 μm and a scanning depth of 2 mm.

### IMAGE DATA ANALYSIS:

Raw FPF images were securely transmitted and analyzed by C.R. from OcuSciences using proprietary algorithms that utilize a convolutional network to segment an optic disc annular region of interest (ROI) based on the infrared images. The FPF measurements were obtained within an annulus of inner diameter 0.5x and outer diameter 1.0x the disc rim, as segmented from the co-registered infrared image. We excluded low-quality images, such as those with cropped structures of interest, out-of-focus images, or those with artifacts and shadows. The FPF image was first adjusted for lens autofluorescence and signal attenuation by the lens or intraocular transmission. An algorithmically generated mask was also applied to the optic disc image to reduce measurement bias from vessels, whose opacity obscures the FPF signal. Global (“G”) optic disc FPF was calculated as averaged FPF in the entire optic disc annulus ROI. FPF average intensities within each Garway-Heath sectors around the optic disc were also calculated, with the origin aligned to the disc-fovea axis. To accommodate a wide signal dynamic range, the optic disc FPF values are expressed in dB, a Log10-based scale. We also calculated the average macular FPF score, expressed in grayscale units (GSU), on a linear scale.

We measured OCT pRNFL thickness using automated segmentation by the company’s algorithm at a 3.46 mm diameter circle around the optic disc. The thickness of the mGCC (combined thickness of the ganglion cell layer and inner plexiform layer) was also automatically segmented by the company’s algorithm in an elliptical annulus centered on the fovea (vertical outer and inner radii of 2.0 and 0.5 mm and horizontal outer and inner radii of 2.4 and 0.6 mm, respectively).

### STATISTICAL ANALYSIS:

Statistical analyses were conducted using Python (version 3.9.6; Python Software Foundation). Descriptive statistics were generated for each group, reporting continuous variables as means with SEs and categorical variables as frequencies and percentages. Welch’s t-test was used to analyze continuous demographic variables across groups, while chi-square tests of independence were used for categorical variables. Group differences in ophthalmic parameters were examined with mixed-model analyses of variance (ANOVAs), using group as the between-subjects factor and incorporating the effects of age and optic nerve sector as within-subjects factors. Statistical significance was set at *P* < .050, except in cases of multiple comparisons, where Bonferroni corrections were applied.

## RESULTS

### ELEVATED OPTIC DISC FLAVOPROTEIN FLUORESCENCE SIGNAL IN OPTIC DISC DRUSEN:

We performed a cross-sectional study to investigate changes in optic disc and macular FPF in eyes with ODD and healthy controls. Our study cohorts consisted of 53 controls (69 eyes) and 94 ODD participants (157 ODD eyes) diagnosed by a neuro-ophthalmologist and underwent comprehensive ophthalmic assessments (Table 1) (See Methods for inclusion and exclusion criteria). Compared with controls, the ODD group is significantly younger (controls: 7-89 years, median 38.5 years; ODD: 10-78 years, median 51 years) and has a higher proportion of females (controls: 28/53, or 52.8% female; ODD: 67/94, or 71.3% female). Both groups have a higher prevalence of white participants, which did not differ between the groups ( χ^2^ = 0.170, *P* = .680).

**FIGURE 1.**
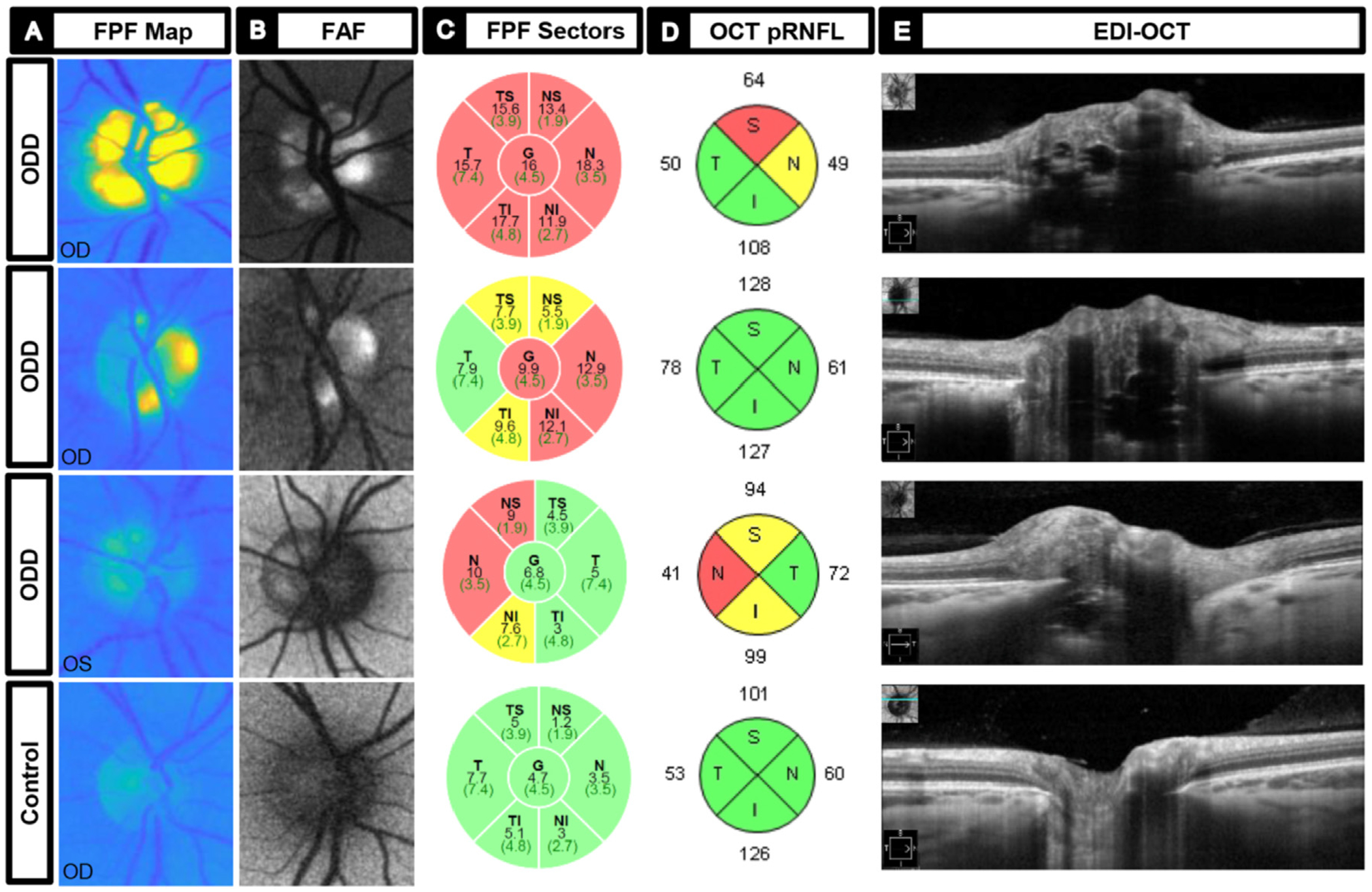
Representative examples showing relatively increased optic disc flavoprotein fluorescence (FPF) signal in ODD eyes compared with control and confirmation of ODD on autofluorescence (FAF) imaging and on enhanced depth imaging optical coherence tomography (EDI-OCT). (A) Optic disc FPF map. (B) Optic disc FAF. (C) Optic disc FPF global and sector values. (D) peripapillary retinal nerve fiber layer (pRNFL) sector measurements. (E) EDI-OCT B scans through the disc. (1st row) Right eye (OD) of a 29-year-old female with severe ODD in all sectors. (2nd row) Right eye of a 22-year-old female with severe ODD in the nasal, superior, and inferior sectors. (3rd row) Left eye (OS) of a 33-year-old male with buried ODD. (4th row) Right eye of a 22-year-old female control.

Compared with healthy controls, eyes with superficial or buried ODD exhibit a higher optic disc FPF signal but not macular FPF (Figure 1, Table 1). Representative examples demonstrate that the location and severity of ODD can be easily visualized on disc FPF, and that the areas with the most intense FPF signals correspond well with hyperautofluorescence on FAF and the typical appearances of ODD on EDI-OCT (Figure 1, Supplementary Figure 2). In pediatric ODD, which can be challenging to image due to buried drusen and a relatively thicker pRNFL, disc FPF can also quantify buried ODD, which are similar in location and severity to those of EDI-OCT B-scans (Supplementary Figure 2). Unlike FAF and EDI-OCT B-scans, which only provide qualitative imaging, the optic disc FPF signal can be used to calculate global FPF scores for the eye or sectoral scores per Garway-Heath map (Figure 1C).

### QUANTIFICATION OF DISEASE BURDEN USING OPTIC DISC FPF SCORE:

Compared with controls, global optic disc FPF was significantly elevated in the ODD group (ODD: 10.82 ± 0.36 dB, control: 4.58 ± 0.21 dB, *P* < .001). In contrast, macular FPF did not differ significantly between the ODD and control groups (ODD: 20.6 ± 0.44 gray scale unit (GSU), control: 20.78 ± 0.61 GSU, *P* = .148). The ODD group also had significantly worse visual acuity (*P* = .036), lower static perimetry mean deviation (MD) (*P* < .001), and OCT pRNFL and mGCC thicknesses (*P* < .001 for both) (Table 1). These findings highlight the structural and functional alterations associated with ODD, which are consistent with an optic neuropathy.

**FIGURE 2.**
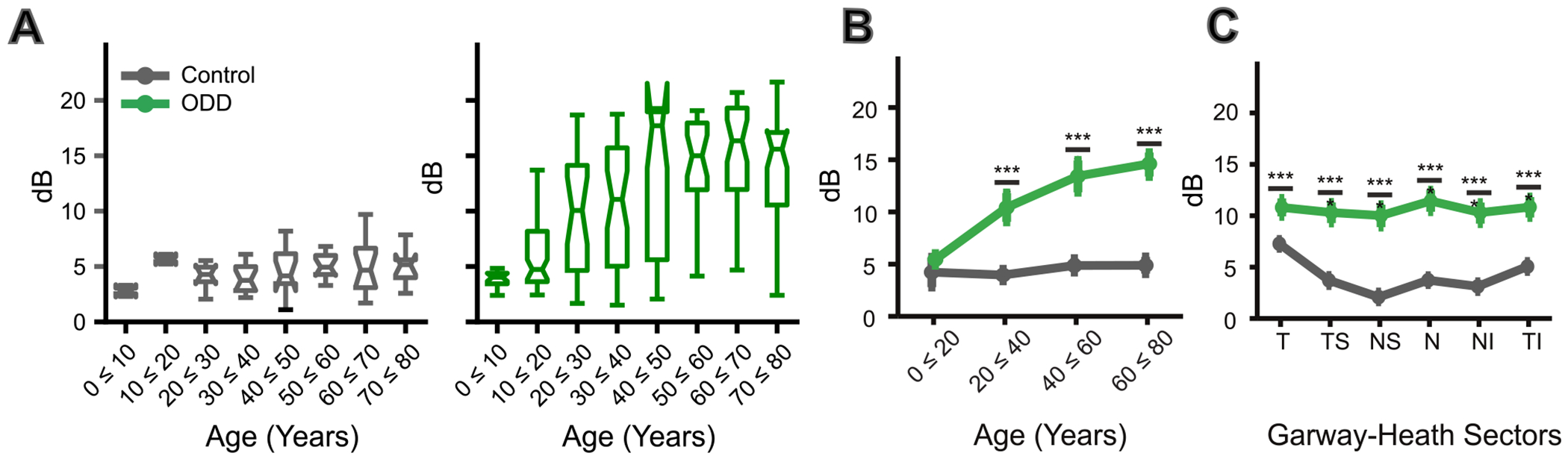
Age-dependent optic disc FPF changes differ between ODD eyes and controls. (A) Box-and-whisker plots illustrating optic disc FPF scores for control and ODD groups stratified by 10-year age intervals. Boxes represent the IQR (IQR), with notches indicating the 95% CIs for the median. FPF values demonstrate age-dependent increases in ODD patients while remaining relatively constant in controls. (B) Interaction plot illustrating significant between-group differences across age strata. Mixed ANOVA analyses demonstrate that FPF scores are comparable between ODD eyes and controls below age 20 but progressively diverge with significantly elevated values in ODD patients beginning in the third decade (20-40 years). Age was stratified into 20-year intervals for ANOVA analyses due to sample size constraints in certain decades. (C) Sectoral analysis reveals significant interactions between disease status and Garway-Heath sectors. FPF scores are significantly elevated across all sectors in the ODD group relative to controls. Within the ODD cohort, the nasal sector demonstrates maximal FPF values (nasal vs naso-superior: *P* < .001; nasal vs naso-inferior: *P* < .001), corresponding to the established topographical predilection of this condition. In contrast, control subjects exhibit maximal FPF values in the temporal sector. These findings establish the age-independent utility of FPF as a quantitative measure of mitochondrial stress and its capacity to detect regional optic disc vulnerability. Statistical significance: ns: *P* > .05, *P ≤ .05, **P ≤.01, ***P ≤.001, ****P ≤.0001. Note: Mixed ANOVA analysis of the main effects of diagnosis and age group on optic disc FPF revealed significant differences. Post hoc Tukey HSD pairwise comparisons across age groups demonstrated significant differences between control and ODD subjects in all age groups 20 years and above.

Segregating disc FPF scores by sectors revealed that ODD eyes have significantly elevated FPF scores across all disc sectors compared to controls (Figure 2C). Within the ODD group, the nasal sector has the highest disc FPF values and is significantly higher than those of the naso-superior and naso-inferior sectors (*P* < .001 for both). This distribution pattern corresponds with the known anatomical predilection of the nasal and superior sectors for the development of ODD and hyperautofluorescence in FAF. In the control group, the temporal sector has the highest disc FPF scores, which is significantly higher than all other sectors (*P* < .001). This relatively higher temporal FPF value in control optic discs may be related to the high density of mitochondria in the papillomacular bundle. Overall, elevated disc FPF is likely due to the combination of ODD autofluorescence signal and intra-axonal mitochondrial stress.

### ELEVATED OPTIC DISC FPF IN ODD PATIENTS BEYOND 20 YEARS OF AGE:

Segregation of optic disc FPF scores by age reveals that control eyes have low disc FPF scores that are relatively stable across decades. The ODD eyes within the first two decades of life have similar disc FPF scores compared to those of the controls (Figure 2A). Between ages 20 and 80, stratified in 20-year intervals, ODD eyes have significantly elevated disc FPF scores compared to the controls (Figure 2B). A 2-way between-subjects ANOVA with disease group and age as independent variables shows a significant main effect of disease group (F(1, 339) = 160.159, *P* < .001) and age stratification (F(3, 339) = 59.504, *P* < .001) and a significant interaction between these two factors (F(3, 339) = 4.438, *P* = .004) (Supplementary Table 1). The presence of a heightened optic disc FPF signal in ODD eyes of older patients, which is absent in the optic disc of age-matched healthy individuals, indicates that FPF signal variations occur independent of normal aging processes and are attributable to disease burden.

### ODD EYES WITH VISUAL FIELD LOSS HAVE HIGHER DISC FPF AND LOWER OCT MEASUREMENTS:

**FIGURE 3.**
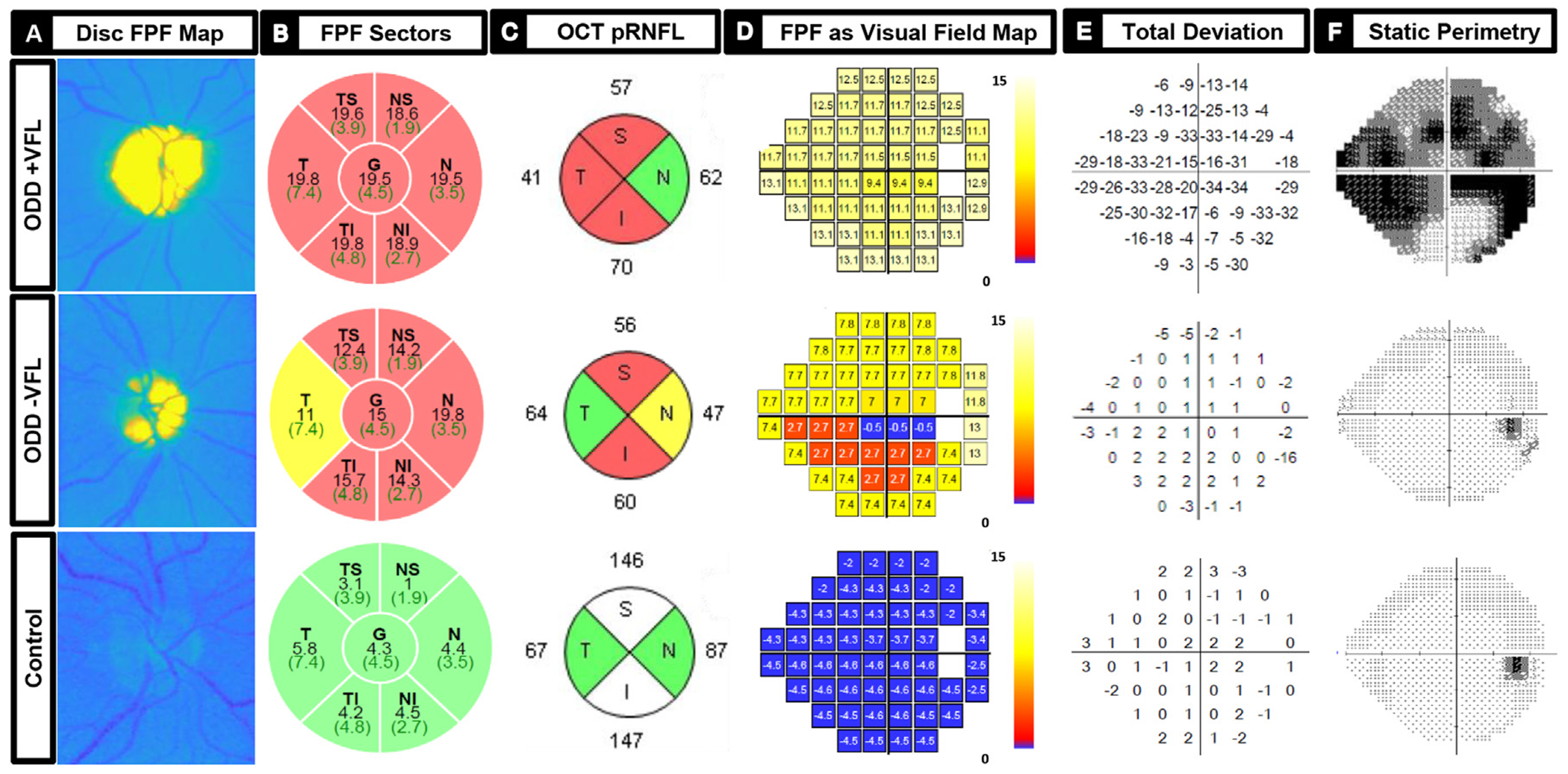
Representative ODD eyes showing increased optic disc flavoprotein fluorescence (FPF) (A, B), OCT pRNFL (C), altered optic disc FPF sector scores represented as visual field per Garway-Heath map (D), and static perimetry gray scale map (E, F) compared with a healthy control eye. (Top row) 62-year-old female with severe ODD and generalized visual field loss (visual acuity 20/25 OU, static perimetry mean deviation –19.81 dB). Optic disc FPF was elevated in all quadrants. (Middle row) 56-year-old female with moderate ODD and enlargement of blind spot (visual acuity 20/25 OD, mean deviation 0.09 dB). FPF is most elevated in the superior, nasal, and inferior quadrants. (Bottom row) 59-year-old female with good vision and optic nerve (visual acuity 20/20 OU, mean deviation 0.56 dB). All eyes shown are right eyes.

**FIGURE 4.**
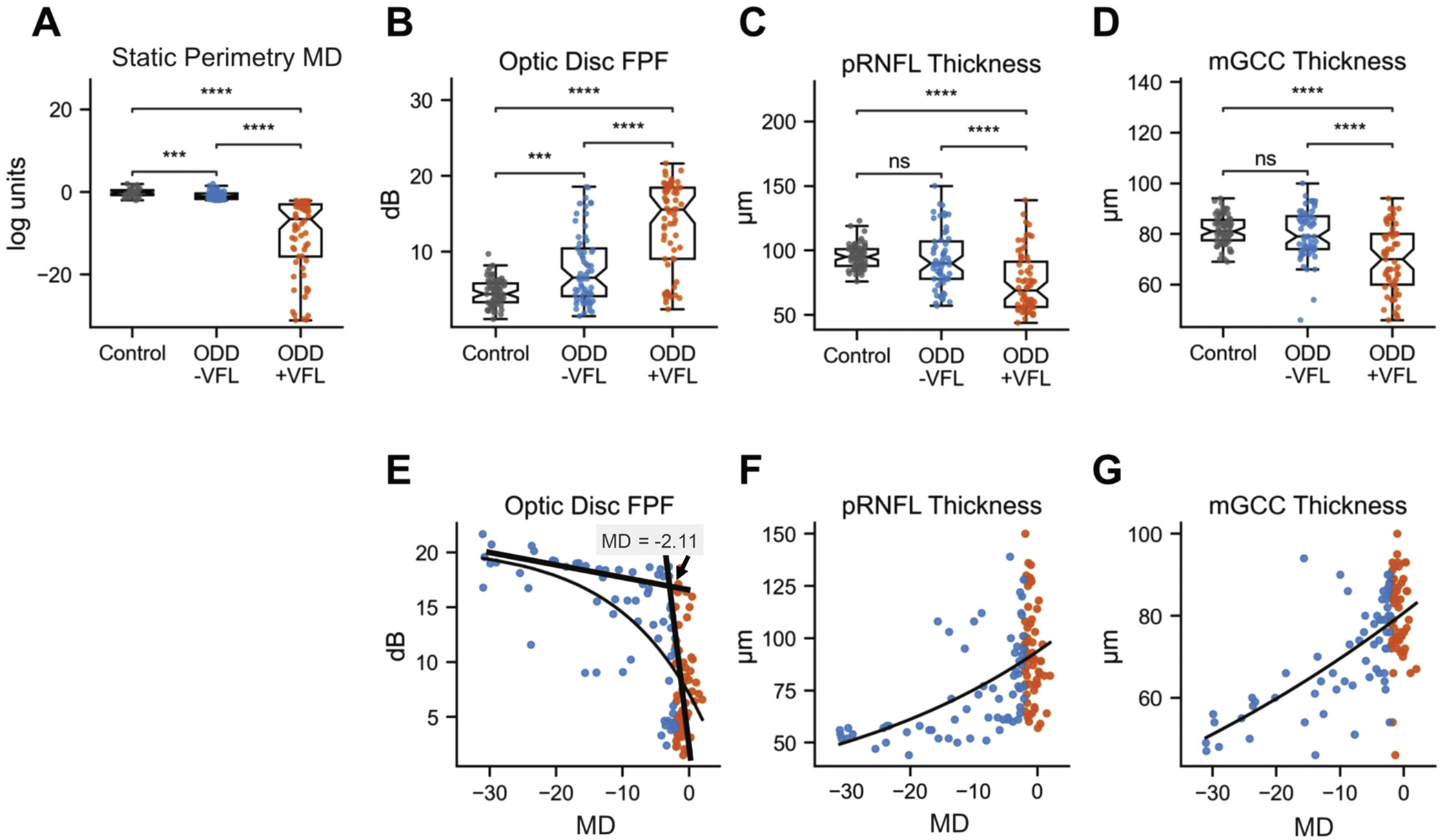
Comparative analysis of functional and structural parameters in ODD patients and controls. (A-D) Box-and-whisker plots illustrating (A) static perimetry mean deviation, (B) optic disc FPF, (C) pRNFL thickness, and (D) mGCC thickness measurements across 3 cohorts: control subjects (gray), ODD patients without visual field loss (ODD-VFL) (blue), and ODD patients with visual field loss (ODD + VFL) (orange). Boxes represent the IQR (IQR), with whiskers extending to the minimum and maximum values within 1.5 × IQR. Statistical analyses demonstrate significantly elevated optic disc FPF in ODD + VFL eyes compared to both controls and ODD-VFL eyes. ODD + VFL subjects exhibited significantly reduced OCT measurements (pRNFL and mGCC) with corresponding decreased mean deviation on static perimetry. (E-G) Scatter plots depicting the relationship between static perimetry mean deviation and key ophthalmic parameters in ODD eyes with and without visual field loss. Nonlinear models characterize the relationship between functional and structural parameters (thinner black lines). Implementation of a sigmoid-exponential model identified an inflection point (black arrow) for optic disc FPF corresponding to − 2.11 dB mean deviation (thicker black lines). *Abbreviations:* FPF = flavoprotein fluorescence; mGCC = macular ganglion cell complex; MD = mean deviation; pRNFL = peripapillary retinal nerve fiber layer. Statistical significance is indicated as follows: ns: *P* > .05, *P ≤.05, **P ≤.01, ***P ≤.001, ****P ≤.0001.

We analyzed the relationship between static perimetry MD and three imaging measurements: optic disc FPF, pRNFL, and mGCC (Figures 3 and 4). We found that ODD eyes with visual field loss (ODD + VFL, MD < −2.0 dB) have significantly higher disc FPF and thinner pRNFL and mGCC compared with ODD eyes without visual field loss (ODD-VFL, MD ≥ −2.0 dB) (*P* < .001 for all, Table 2 and Supplementary Table 2). The ODD + VFL group has significantly lower pRNFL thickness compared to the ODD-VFL group and to controls (ODD + VFL: 74.9 ± 2.8 μm, ODD-VFL: 93.0 ± 2.9 μm, controls: 95.25 ± 1.1 μm; *P* < .001 for all, Table 2 and Supplementary Table 2). A similar pattern was observed for mGCC thickness, with the ODD + VFL group showing significantly lower values relative to the ODD-VFL group and with controls (ODD + VFL: 69.9 ± 1.6 μm, ODD-VFL: 79.9 ± 1.3 μm, controls: 81.6 ± 0.7 μm; *P* < .001 for all, Table 2 and Supplementary Table 2)

While the ODD-VFL group has significantly higher disc FPF compared with controls (*P* < .001), there is no difference in pRNFL and mGCC between these two groups (Table 2). The ODD-VFL group has similar pRNFL thickness compared to controls (*P* = .78). A similar pattern was observed for mGCC thickness (*P* = .6). Collectively, our findings demonstrate significant differences in visual function and structure between ODD eyes with and without visual field loss.

### OPTIC DISC FPF HAS A NONLINEAR RELATIONSHIP WITH STATIC PERIMETRY MEAN DEVIATION:

Figure 4E demonstrates a negative correlation between optic disc FPF and static perimetry MD, where ODD eyes with more visual field loss have higher disc FPF scores. To determine the point of inflection, two additional models were applied to the optic disc FPF data. The intersection between the flattest (superior aspect of the figure) and steepest (right aspect of the figure) lines of best fit identifies a marked increase in disc FPF at approximately MD of −2.11 dB. The comparison of MD, pRNFL, and mGCC thicknesses reveals nonlinear positive correlations, where ODD eyes with more pronounced visual field deficits have thinner pRNFL and mGCC (Figure 4F, G). Quantitative assessment of the relationship between the structural and metabolic parameters revealed significant associations (Supplementary Figure 3A).

### OPTIC DISC FPF IS NEGATIVELY CORRELATED WITH OCT MEASUREMENTS:

In ODD patients, disc FPF scores demonstrate a strong negative correlation with pRNFL thickness (*r* = −0.78) and a moderate negative correlation with mGCC thickness (*r* = −0.62) (Supplementary Figure 3A). Cluster analysis further confirms this inverse relationship, with control subjects exhibiting greater structural integrity but lower FPF values compared to ODD patients (Supplementary Figure 3B). The model using optic disc FPF demonstrates the lowest root mean square error among the three ophthalmic parameters evaluated (Supplementary Table 3). Collectively, these findings suggest that both optic nerve and macular structural alterations are strongly associated with the degree of visual dysfunction in patients with ODD.

### MULTIPLE OPHTHALMIC PARAMETERS SEGREGATE ODD AND CONTROL GROUPS:

Comparative analyses of ophthalmic parameters (Table 3 A) between controls and ODD shows that the optic disc FPF demonstrates a substantial negative effect size (Cohen’s *d* = 1.129, power = 1.0), indicating significantly elevated values in ODD compared to controls. The mGCC thickness exhibited a moderate to large effect size (Cohen’s *d* = 0.562, power = 0.96708), suggesting a meaningful distinction between the ODD and control groups. Static perimetry MD also shows a moderate effect size (Cohen’s *d* = 0.746, power = 0.95915), further supporting differences between cohorts. In contrast, macular FPF, LogMAR visual acuity, and IOP demonstrate small effect sizes (Cohen’s d ranging from 0.025 to 0.238) with low to moderate statistical power (0.05337-0.35794), indicating minimal or uncertain group differences. These findings highlight significant disparities in optic disc function and macular ganglion cell complex measurements, while other parameters demonstrate less pronounced or inconclusive variations.

### POWER ANALYSIS SHOWS OPTIC DISC FPF SCORE SEGREGATED ODD EYES WITH AND WITHOUT VISUAL FIELD LOSS:

A comparative analysis between the ODD + VFL and ODD-VFL groups (Table 3B) reveals a pronounced effect size for optic disc FPF (Cohen’s *d* = 1.069, power = 0.99997), indicating substantial differences between these 2 clinical subgroups. The mGCC thickness and pRNFL thickness show moderate to large effect sizes (Cohen’s *d* = 0.872 and 0.797, respectively) with high statistical power (0.99725 and 0.99366, respectively), reinforcing notable structural differences between ODD eyes with and without visual field loss. Conversely, macular FPF, LogMAR visual acuity, and IOP display small effect sizes (Cohen’s d from−0.157 to 0.242) and low to moderate power (0.12446 to 0.2721), suggesting limited discriminatory capacity. All effect sizes are reported in absolute values. In summary, optic disc FPF, mGCC, and pRNFL show high statistical power, while other parameters, such as macular FPF, visual acuity, and IOP, exhibit weaker or less definitive differences between ODD eyes with and without visual field loss.

## DISCUSSION

The current most common imaging modalities used to diagnose and monitor patients with ODD include *en face* FAF^28^ and EDI-OCT 97 B-scans through the optic disc,^12,28^ which provide *qualitative* assessments of disease burden in ODD. Using FPF imaging, we show that eyes with ODD exhibit significantly higher global optic disc FPF, but not macular FPF, compared with healthy controls, and that disc FPF can quantify disease burden in adult and pediatric patients across a wide range of disease severity. In eyes with focal ODD deposits, sectoral disc FPF can quantify regional disease burden. FPF can also detect buried drusen that may not be obvious on FAF imaging. Thinning of the pRNFL and mGCC layer is commonly seen in patients with ODD,^29,30^ and our study shows a moderately negative correlation between optic disc FPF with pRNFL and mGCC, signifying increased global optic disc FPF with thinning of the pRNFL and mGCC in patients. While disc FPF, pRNFL, and mGCC all have high statistical power in segregating patients with no visual field loss from those with visual field loss, optic disc FPF may be more specific. In comparison to mGCC and pRNFL, the majority of the signal likely comes from the hyperautofluorescent calcified deposits prior to the development of changes in pRNFL and mGCC.^22^ The benefits of incorporating optic disc FPF into neuro-ophthalmic practice include: quantification of disease burden to better assess disease severity at baseline, improved participant selection for clinical trials, and better understanding of the relationship between disease severity and vision loss.

We investigated the optic disc FPF scores between 10 and 80 years of age and found that ODD eyes between 10 and 20 years of age exhibit relatively increasing optic disc FPF and that the high disc FPF levels occur by age 30. While this is not a longitudinal study, this pattern is consistent with the second and third decades of life as a high risk for progression of ODD. An increased disc FPF score can be due to multiple factors, including increased size of ODD, ODD becoming more superficial, loss of overlaying pRNFL, or increased axonal metabolic stress at the optic disc. Age-related increase in ODD size and number, along with age-related progressive optic atrophy in ODD patients, has been reported in previous studies.^31–33^ A longitudinal study with detailed morphometric analysis is needed to further investigate the slope of change in global or sectoral disc FPF scores compared with other ophthalmic parameters over time, especially in patients in the first 3 decades of life, when these values may change more rapidly due to a combination of ODD and evolving optic neuropathy. Although we do not currently understand the factors that lead to acceleration of vision loss in ODD, patients should be screened for risk factors of NAION, since this is a common cause of vision loss in ODD^34^, and some of the risk factors can be mitigated.

Sectoral analysis of disc FPF in ODD eyes vs. healthy controls reveals that ODD eyes have significantly higher disc FPF values in all sectors compared with controls. In optic neuropathies, the temporal disc sector typically has the highest level of metabolic stress and the highest disc FPF values.^35^ Amongst the ODD eyes, FPF scores are relatively higher in the nasal, naso-superior, and naso-inferior sectors. This aligns with prior publications describing ODD as most commonly occurring in the nasal and superior sectors.^36–40^

Perimetry is currently the best measurement of visual function in ODD, as patients with ODD typically have preserved visual acuity. On comparing ODD eyes with and without visual field loss (defined as MD <−2.0 dB), we show that optic disc FPF is significantly higher in the group with visual field loss, aligning with previous studies^21^ depicting a negative correlation between visual field mean deviation and optic disc FPF. Detailed analysis reveals that there is a nonlinear relationship between optic disc FPF and static perimetry MD. At MD <−2.0 dB, there is a wide range of disc FPF scores (2 to 20 dB). This large range of ODD disease burden without visual field loss has been described previously^41,42^ and indicates that remarkably, disease burden can increase dramatically, likely over years, without visual field loss. However, at static perimetry MD worse than −2.0 dB, there is an exponential relationship between optic disc FPF and MD, indicating that at a threshold of disease severity, which may vary among different patients, as disc FPF score increases, the severity of visual field loss can develop rapidly. Given that static perimetry is a subjective measurement that may be highly variable, especially in those with almost no visual field loss, repeat testing is often needed to determine whether the patient is truly developing vision loss. Future studies comparing disc FPF severity and corresponding visual field representation using the Garway-Heath map may help increase confidence in diagnosing new visual field loss. A higher disc FPF may reflect an increase in the severity of axonopathy or an increase in the size of ODD. Increased optic disc FPF score may be due to increased axonopathy, and there is evidence that FPF imaging may reflect oxidative stress before structural damage.^22^ Optic disc FPF in combination with EDI-OCT and OCT pRNFL and GCC can help distinguish increased drusen deposit vs increased axonopathy over time and increase clinician confidence in the progression of the optic neuropathy. In summary, future clinical trials in ODD are best designed using a combination of visual function assessments and ophthalmic imaging, and an important next step is to determine whether optic disc FPF can be a novel outcome measure for ODD since use of imaging measurement of geographic atrophy lesion as primary end point has greatly improved clinical trial in age-related macular degeneration and facilitated discovery of new therapies.^43^

There are two important technical details in our study that warrant further discussion: the comparison of FPF vs. FAF and the use of optic disc (papillary) FPF rather than peripapillary FPF. FPF imaging is a type of autofluorescence imaging. The healthy human optic disc is normally relatively dark on both blue and green-light FAF imaging^44^, whereas it is typically slightly brighter than the surrounding peripapillary tissue in FPF images. There is an overlap of the excitation and emission filters used to perform FPF and conventional blue FAF imaging (Supplementary Figure 1), but the FPF imaging with narrower spectral filters is designed to capture endogenous flavoproteins^45^ while the blue FAF imaging is designed to capture a wider range including predominantly retinal lipofuscin (autofluorescence in the yellow-orange-red range, 540 nm to more than 700 nm with a peak at 600 nm). FPF imaging using Oc-uMet Beacon is performed using a blue LED light with an additional bandpass narrow filter centered at 471 nm, and a very narrow emission filter at green 530 nm. These filter sets are designed based on previous imaging studies of endogenous flavoproteins, which are primarily located in the mitochondria^14^. Blue FAF imaging with the Heidelberg Spectralis uses a solid-state 488 nm blue light laser for excitation and a broad emission filter of 570 to 780 nm. Green FAF imaging with Optomap utilizes a 532 nm green light laser for excitation and a broad emission filter of 480 to 800 nm. More studies are needed to better understand the components of the autofluorescence signal in ODD and the change over time due to axonopathy vs enlargement of deposits containing hydroxyapatite crystals.

In this study, we focused on the papillary rather than the peripapillary FPF because the papillary FPF is dominated by signal from the axons, while the peripapillary FPF signal is diluted by contributions from the entire thickness of the peripapillary retina, including the lipofuscin from retinal pigment and contributions from Bruch’s membrane. In contrast to optic disc FPF, the current most common way to quantify the unmyelinated retinal ganglion cell axons uses OCT to segment the thickness of the peripapillary RNFL. As an epifluorescence measurement, FPF cannot be segmented to identify the contribution from a specific retinal layer.

Limitations of our study include the lack of longitudinal data points, which restricts our ability to understand the evolution of the FPF signal in relation to disease progression or for long-term monitoring. Future longitudinal studies can help understand the change in optic disc FPF score as a result of aging and the natural history of progression of ODD, optic neuropathy, and vision loss. Our study is also limited by the lack of age or gender matching between the ODD and control groups. Despite the two groups not being matched for age or gender, our data shows that there is no relationship between disc FPF values and age or gender in the control population. Additionally, our study has limited data from the population in the first three decades of life to assess the relationship of FPF with respect to age effectively. This can be addressed by a future prospective study with age-matched populations.

## CONCLUSION

In summary, we demonstrate that compared with healthy control eyes, ODD eyes exhibit significantly elevated optic disc FPF scores by the third decade of life. Optic disc FPF has high statistical power to segregate not only control and ODD eyes but also ODD eyes with and without visual field loss. The global optic disc FPF score is a good single quantitative representation of ODD disease burden and can be used to monitor changes over time. Optic disc FPF is a highly promising imaging measurement of disease burden that should be further explored in future prospective studies.

## Supplementary Material

supplementary material

Supplemental Material available at AJO.com.

## Figures and Tables

**TABLE 1.** Patient Demographic and Ophthalmic Data for Control and ODD Groups

Parameters	Controls	ODD	Statistical Analysis
Sample size	53 (69 eyes)	94 (157 eyes)	
Age in years	51 (10-78)	38.5 (7-89)	*t* = 2.911, *P* = .004^a^
Gender			
Female	28 (38 eyes)	67 (114 eyes)	X^2^ = 4.270, *P* = .039^b^
Male	25 (31 eyes)	27 (43 eyes)	
Ethnicity			
White	44 (56 eyes)	74 (124 eyes)	X^2^ = 0.170, *P* = .680^b^
Hispanic or Latino	1 (2 eyes)	5 (7 eyes)	
Asian	6 (8 eyes)	6 (10 eyes)	
Pacific Islander	0 (0 eyes)	1 (2 eyes)	
Other or declined to state	2 (3 eyes)	12 (14 eyes)	
Ophthalmic measurements			
Optic disc FPF (dB)	4.58 ± 0.21	10.82 ± 0.36	*U* = 15 408.5, *P* < .001^c^
Macular FPF (GSU)	20.78 ± 0.61	20.6 ± 0.44	*U* = 8564.0, *P* = .148^c^
Static perimetry MD (dB)	−0.12 ± 0.18	−5.91 ± 0.51	*U* = 1079.0, *P* < .001^c^
LogMAR (log units)	0.02 ± 0.01	0.08 ± 0.02	*U* = 9289.5, *P* = .036^c^
pRNFL thickness (μm)	95.25 ± 1.1	83.88 ± 1.53	*U* = 6215.5, *P* < .001^c^
mGCC thickness (μm)	81.61 ± 0.7	75.14 ± 0.77	*U* = 6357.0, *P* < .001^c^
IOP (mm Hg)	15.78 ± 0.5	15.21 ± 0.26	*U* = 5689.5, *P* = .253^c^

Abbreviations: ODD = optic disc drusen; FPF = flavoprotein fluorescence; GSU = grayscale units; MD = mean deviation; LogMAR = logarithm of the minimum angle of resolution; pRNFL = peripapillary retinal nerve fiber layer; mGCC = macular ganglion cell complex; IOP = intraocular pressure.

a Age was analyzed using Welch’s t-test.

b Gender (proportion of male to female subjects in clinical groups) and ethnicity (proportion of white to non-white subjects) were analyzed using chi-squared tests of independence.

c Ophthalmic measurements were analyzed using the Mann-Whitney U tests.

**TABLE 2.** One-way ANOVA and Tukey HSD Pairwise Comparisons for Ophthalmic Variables With a Significant^a–c^ Main Effect of Diagnosis Group (−VFL, +VFL, and Control)

Ophthalmic Measurements	df1	df2	F	*P*	np^2^	A	B	Diff	S.E.	*P*
Optic Disc FPF (dB)	2	194	72.149	<.001	0.427	Control	+VFL	−8.943	0.751	<.001
						Control	−VFL	−3.279	0.767	<.001
						+VFL	−VFL	−5.664	0.777	<.001
Static Perimetry MD (dB)	2	155	52.922	<.001	0.406	Control	+VFL	20.371	3.307	<.001
						Control	−VFL	2.217	3.375	.789
						+VFL	−VFL	18.154	3.399	<.001
pRNFL Thickness (μm)	2	192	22.474	<.001	0.19	Control	+VFL	10.424	1.303	<.001
						Control	−VFL	0.763	1.32	.832
						+VFL	−VFL	9.661	1.063	<.001
mGCC Thickness (μm)	2	184	25.728	<.001	0.219	Control	+VFL	11.694	1.742	<.001
						Control	−VFL	1.663	1.757	.612
						+VFL	−VFL	10.031	1.797	<.001

Abbreviations: −VFL = optic disc drusen patients without visual field loss; +VFL = optic disc drusen patients with visual field loss.

a Macular FPF (GSU): No significant differences in Macular FPF were observed between the groups (F(2193) = 1.158, *P* = .316, *η*^2^ = 0.012), indicating similar macular metabolic activity groups.

b LogMAR: Visual acuity as measured by LogMAR did not differ significantly between groups (F(2168) = 2.903, *P* = .058, *η*^2^ = 0.033), though this result approaches the threshold of statistical significance.

c IOP (mm Hg): Intraocular pressure showed no significant differences between the groups (F(2155) = 0.757, *P* = .471, *η*^2^ = 0.01), suggesting comparable pressure values across all conditions.

**TABLE 3.** Power Calculated Using the Effect Size (Cohen’s d) of Differences in Ophthalmic Measurements Between **(A)** ODD and Control Eyes and Between **(B)** ODD Eyes With and Without Visual Field Loss

A	Ophthalmic Measurements	Control	ODD	Cohen’s d (abs)	Power
	Optic disc FPF (dB)	69	157	1.129	1
	mGCC thickness (μm)	67	148	0.562	0.967
	Static perimetry MD (dB)	31	127	0.746	0.959
	pRNFL thickness (μm)	68	157	0.482	0.911
	LogMAR	66	145	0.238	0.358
	IOP (mm Hg)	54	130	0.146	0.146
	Macular FPF (GSU)	68	157	0.025	0.053
**B**	**Ophthalmic measurements**	**ODD-VFL**	**ODD+VFL**	**Cohen’s d (abs)**	**Power**
	Optic disc FPF (dB)	66	61	1.069	0.999
	mGCC thickness (μm)	61	59	0.872	0.997
	pRNFL thickness (μm)	66	61	0.797	0.994

Abbreviations: ODD = optic disc drusen; FPF = flavoprotein fluorescence; GSU = grayscale units; MD = mean deviation; LogMAR = logarithm of the minimum angle of resolution; pRNFL = peripapillary retinal nerve fiber layer; mGCC = macular ganglion cell complex; IOP = intraocular pressure.

## Abbreviations and Acronyms

ODD: Optic disc drusen
OCT: optical coherence tomography
LogMAR: logarithm of the minimum angle of resolution
EDI-OCT: enhanced depth imaging-optical coherence tomography
FAF: fundus autofluorescence
FPF: flavoprotein fluorescence
mGCC: macular ganglion cell complex
HVF: Humphry visual field 24-2
pRNFL: peripapillary retinal nerve fiber layer
RPE: retinal pigment epithelium
VFL: visual field loss
IOP: intraocular pressure
