# Supplementary material for: Optic Disc Flavoprotein Fluorescence Imaging as a Novel Method to Quantify Disease Burden in Optic Disc Drusen
Source: Am J Ophthalmol. Author manuscript; Available in PMC 2026 Jul 27. (PMC13406388; doi:10.1016/j.ajo.2025.11.018)
Supplement: supplementary material [file NIHMS2191907-supplement-supplementary_material.docx]

| **Table 1 Supplementary.** Mixed ANOVA analyzing the main effect of diagnosis and age group on optic disc FPF. Post hoc Tukey HSD pairwise comparisons for each age group revealed significant differences between control and ODD subjects in age groups 20 years and above. | | | | | | | | |
| --- | --- | --- | --- | --- | --- | --- | --- | --- |
| **A** | Variable | SS | df | MS | F | p | | np2 |
|  | Diagnosis | 3185.868 | 1 | 3185.868 | 160.159 | < .001 | | 0.321 |
|  | Age | 3550.942 | 3 | 1183.647 | 59.504 | < .001 | | 0.345 |
|  | Diagnosis * Age | 264.847 | 3 | 88.282 | 4.438 | 0.004 | | 0.038 |
|  | Residual | 6743.369 | 339 | 19.892 |  |  | |  |
| **B** | Age Group | Control n (Mean ± SD) | ODD n (Mean ± SD) | T | df | p | BF10 | hedges |
|  | 0 ≤ 20 | 4 (4.230 ± 1.738) | 45 (5.344 ± 2.538) | -1.1754 | 4.2336 | 0.3017 | 0.685 | -0.4395 |
|  | 20 ≤ 40 | 19 (4.021 ± 1.268) | 35 (10.017 ± 5.947) | -5.8001 | 39.1479 | < .001 | < .001 | -1.2379 |
|  | 40 ≤ 60 | 11 (5.226 ± 1.996) | 30 (13.614 ± 5.934) | -7.7123 | 34.3509 | < .001 | < .001 | -1.8739 |
|  | 60 ≤ 80 | 9 (3.692 ± 1.588) | 43 (14.675 ± 5.142) | -10.8586 | 60.7745 | < .001 | < .001 | -2.2141 |

| **Table 2 Supplementary.** Descriptive statistics of ophthalmic measurements found in each diagnosis subgroup. | | | | | | | |
| --- | --- | --- | --- | --- | --- | --- | --- |
| Ophthalmic Measurements | Group | N | Mean | Median | S.E. | Min | Max |
| Optic Disc FPF (dB) | Control | 70 | 4.61 | 4.425 | 0.206 | 1.1 | 9.7 |
|  | -VFL | 61 | 7.889 | 6.58 | 0.609 | 1.51 | 18.55 |
|  | +VFL | 66 | 13.553 | 15.55 | 0.709 | 2.4 | 21.65 |
| Macular FPF (GSU) | Control | 69 | 20.725 | 20.2 | 0.602 | 12 | 34.1 |
|  | -VFL | 61 | 19.685 | 18.2 | 0.874 | 11.2 | 48.8 |
|  | +VFL | 66 | 21.482 | 18.8 | 0.975 | 8.5 | 48.4 |
| Static Perimetry MD (dB) | Control | 31 | -0.123 | -0.04 | 0.183 | -2 | 1.99 |
|  | -VFL | 61 | -0.886 | -1.08 | 0.123 | -2 | 2.01 |
|  | +VFL | 66 | -10.547 | -6.595 | 1.129 | -31.07 | -2.08 |
| LogMAR | Control | 66 | 0.023 | 0 | 0.008 | 0 | 0.301 |
|  | -VFL | 54 | 0.043 | 0 | 0.014 | 0 | 0.477 |
|  | +VFL | 51 | 0.073 | 0 | 0.022 | 0 | 0.698 |
| pRNFL Thickness (µm) | Control | 68 | 95.25 | 95 | 1.1 | 76 | 123 |
|  | -VFL | 61 | 93.033 | 90 | 2.895 | 57 | 150 |
|  | +VFL | 66 | 74.879 | 69 | 2.821 | 44 | 139 |
| mGCC Thickness (µm) | Control | 67 | 81.612 | 81 | 0.704 | 69 | 94 |
|  | -VFL | 59 | 79.949 | 79 | 1.294 | 46 | 100 |
|  | +VFL | 61 | 69.918 | 70 | 1.644 | 46 | 94 |
| IOP (mm Hg) | Control | 54 | 15.778 | 15 | 0.499 | 9 | 24 |
|  | -VFL | 54 | 15.481 | 15 | 0.523 | 8 | 25 |
|  | +VFL | 50 | 14.84 | 14 | 0.616 | 8 | 32 |
| Abbreviations: ODD = optic disc drusen; FPF = flavoprotein fluorescence; GSU = grayscale units; MD = mean deviation; LogMAR = logarithm of the minimum angle of resolution; pRNFL = peripapillary retinal nerve fiber layer; mGCC = macular ganglion cell complex; IOP = intraocular pressure | | | | | | | |

| **Table 3 Supplementary.** Residuals of exponential line of best fit for each ophthalmic measurement. | | | | |
| --- | --- | --- | --- | --- |
| Ophthalmic Measurements | MAE | MSE | RMSE | R^2^ |
| Optic Disc FPF (dB) | 6.039 | 53.112 | 7.288 | -0.495 |
| pRNFL Thickness (µm) | 21.057 | 685.285 | 26.178 | -0.325 |
| mGCC Thickness (µm) | 11.244 | 208.515 | 14.44 | -0.517 |
| Abbreviations: MAE = Mean Absolute Error, MSE = Mean Squared Error, RMSE = Root Mean Squared Error, R² (R-Squared, Coefficient of Determination).  MAE: Measures the average absolute difference between predicted and actual values. Lower MAE indicates better model accuracy. MSE: Computes the average of squared differences between predictions and actual values, penalizing larger errors more than MAE. RMSE: Square root of MSE, providing an error measure in the same units as the target variable, making interpretation easier. R² (R-Squared): Indicates how well the model explains the variance in the target variable. Values range from 0 to 1, where higher values indicate a better fit. | | | | |


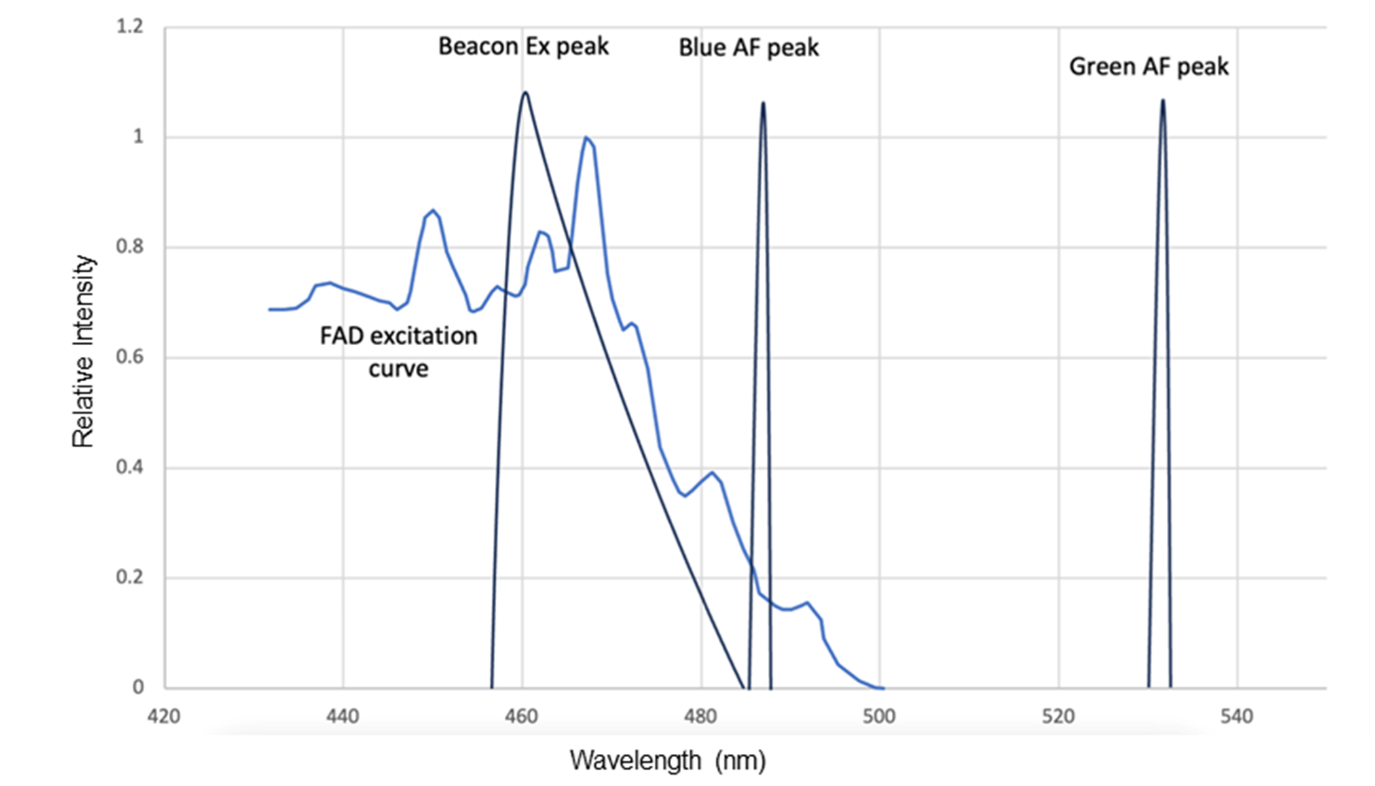


**Supplementary Figure 1.** Relative excitation and emission spectrum of flavoprotein flavin adenine dinucleotide (FAD) by OcuMet Beacon FPF compared with blue- and green-light autofluorescence (AF) imaging. The excitation wavelengths are: FPF at 471nm, blue AF at 488 nm, and green AF at 532 nm.


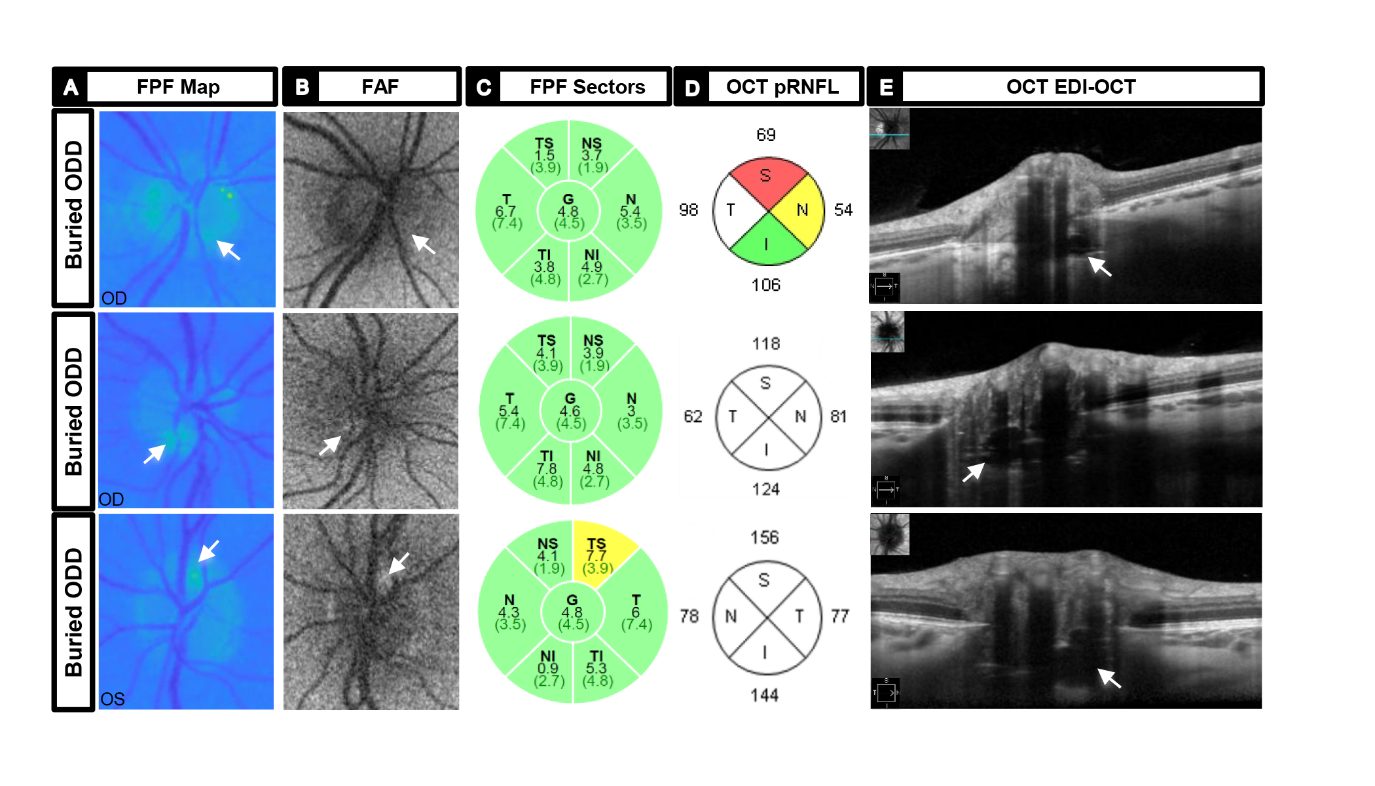


**Supplementary Figure 2.** Eyes with buried optic disc drusen show elevated signal on optic disc flavoprotein fluorescence (FPF) imaging but may be invisible or barely visible on fundus autofluorescence imaging. **(A)** Optic disc FPF map. **(B)** Optic disc green-light fundus autofluorescence (FAF) imaging. **(C)** Optic disc FPF global and sector scores. **(D)** Optical coherence tomography peripapillary retinal fiber layer (OCT pRNFL) sector thicknesses. (E) Enhanced-depth optical coherence tomography (EDI-OCT) B-scans with arrows corresponding to the white arrows in A and B. **(1^st^ row)** Right eye (OD) of a 26-year-old female with pseudo edema due to ODD. **(2^nd^ row)** Right eye of an 8-year-old male with autosomal-dominant ODD. **(3^rd^ row)** Left eye (OS) of a 7-year-old male with autosomal-dominant ODD.


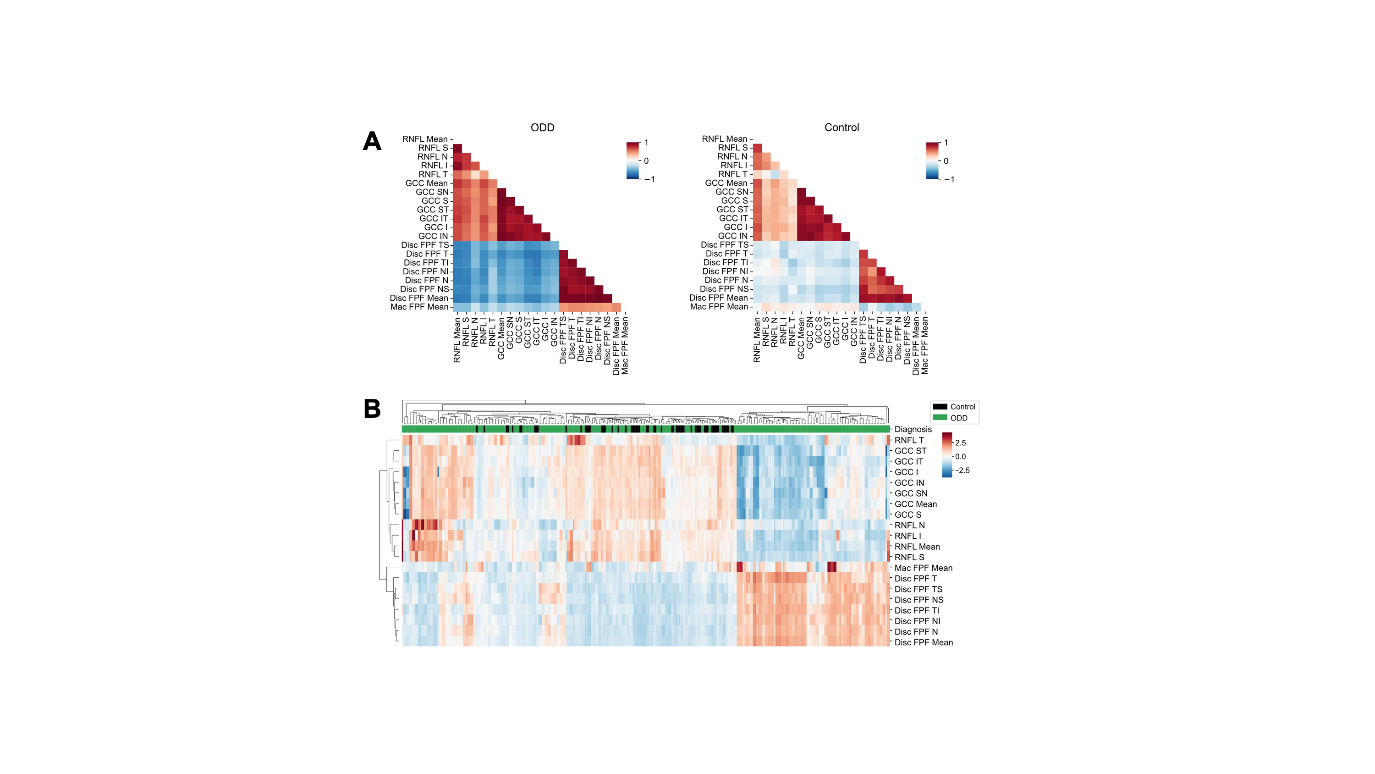


**Supplementary Figure 3. Correlation analysis between FPF values and OCT measurements.** (**A)** Heatmaps illustrating Spearman's rho correlation coefficients between FPF and OCT measurements in ODD patients (left) and control subjects (right). Both groups demonstrated negative correlations between FPF and OCT measurements, with stronger correlations observed in the ODD group compared to controls. Correlation coefficient magnitudes: 0.7-1.0 (high), 0.3-0.7 (moderate), and 0-0.3 (low)**. (B)** Cluster map depicting standardized parameters across groups. Control subjects (grey) exhibited greater structural integrity (higher pRNFL and mGCC thickness) compared to ODD patients, while FPF scores were elevated in ODD patients (green).
